# The Chronic Diseases of Dr. Samuel Hahnemann

**Published:** 1896-04

**Authors:** 


### BOOK NOTICES.

THE CHRONIC DISEASES, THEIR PECULIAR NATURE, AND THEIR HOMŒOPATHIC CURE. By Dr. Samuel Hahnemann. Translated from the second enlarged German edition of 1835 by Prof. Louis H. Tafel, with annotations by Richard Hughes, M. D., edited by Pemberton Dudley, M. D. Philadelphia: Bœricke & Tafel. 1896. 1,600 pages. Price, half morocco, $10.00.

The *Chronic Diseases* of Hahnemann, as translated by Dr. Hempel, is a book known to the profession in the five little, ancient-looking volumes in black cloth binding standing on the shelves of the library of every admirer of the master. These volumes have long been out of print, and have commanded a price far above their original cost, by reason of the increased demand for the work on the part of the rising generation of practitioners.

Recognizing this demand, the firm of Bœricke & Tafel have sought to satisfy it by republishing the work in full. For this purpose they have caused a retranslation to be made and the new translation to be carefully compared with the former one of Hempel.

The result of this investigation reveals that a new translation was urgently needed to correct the errors of the old.

In the Translator's Preface a series of monumental blunders is specifically demonstrated that show that Dr. Hempel's translation was, to say the least, unreliable to a reprehensible degree. The explanation given in the preface to account for this inaccuracy is that Hempel sought to "master the sense of a period, and then embody it in a free manner in the foreign tongue."

There is a graver accusation against Dr. Hempel of a more inexcusable motive for his inaccuracies in translation than the above. This motive was intimated some time since by the editor of this journal in a review of Dr. Hempel's book entitled, *The Science of Homœopathy*, published in The Homœopathic Physician for August, 1895, pages 386 and 387.

In that review it was hinted that Dr. Hempel, by reason of his mistranslations, had aroused toward himself the active resentment and determined hostility of Dr. Lippe. The latter was exceedingly strict and accurate in his reading of Hahnemann's text, a close student of his writings, and perhaps the ablest prescriber on homœopathic principles who ever practiced in Philadelphia.

He was the better qualified to judge of these inaccuracies of translation, because German was his mother tongue, and he had an unusually good command of the English language. He always used the original German text in his own perusals of Hahnemann's works because he did not trust the English rendering.

Intense in his devotion to the Hahnemannian method, successful beyond belief in his practice of it, Dr. Lippe was the implacable enemy of every one who perverted it to any purpose of his own. So it was that he conceived a strong aversion to Dr. Hempel and his writings, and boldly and incessantly denounced him before the classes in the college, in casual conversation with visitors, and in his articles for the journals.

It was Dr. Lippe who made the open accusation that Hempel disputed the truth of much that Hahnemann taught, and that whenever he found a statement which he objected to, he did not hesitate either to throw it out entirely, or else to pervert it to express his own dissenting "view."

These accusations of Dr. Lippe found little credit with the majority of the profession, but they are abundantly verified by the "Translator's Preface" previously mentioned, and indeed the detailed statement of errors there given constitutes in itself an unanswerable indictment, entirely vindicating Dr. Lippe, and at the same time a strong argument for the new translation by Prof. L. H. Tafel.

It may be further said of Dr. Hempel that a man whose intellect was so obtuse that he felt himself perfectly justified in perverting an author's text merely because he did not find it acceptable to his own mind, is hardly fit to be trusted with expounding those doctrines to a college of students nor in other ways setting himself forward as a teacher and leader of thought on so important a subject.

The Materia Medica department was edited by Dr. Richard Hughes, of Brighton, England, who gives the preface to that part of the book, in which he explains the enormous increase in the size of the book.

Dr. Pemberton Dudley, Dean of Hahnemann College, Philadelphia, and President of the American Institute of Homœopathy, was the general editor of the book. He contributed a preface in which he explains Hahnemann's well-known style of involved phrases, and long parentheses and parentheses within parentheses, and declares his intention to maintain a scrupulous fidelity to the original German, regardless of the loss of grace in the English composition. This promise he appears to have fulfilled, and thus has set up an example which is a gratifying contrast to the practice of Hempel as before explained.

After the articles above mentioned, we get Hahnemann's own preface and his famous essay upon the chronic diseases, which has caused so much acrimonious dispute. After that follows the materia medica, which occupies about fourteen hundred pages of the sixteen hundred pages that comprise the book.

This materia medica is, as will be seen, very voluminous.

The symptomatology is not divided off into chapters as we find in the ordinary materia medica. The symptoms are properly grouped for study, but the groups are not labeled.

Every symptom is, however, numbered, and this is, of course, an advantage. The remedies are placed in alphabetical order so that any medicine may be found by glancing at the top of the page.

A table of contents precedes the whole arrangement, and an index follows it. This index is alphabetical and occupies about ten pages.

We have thus a large volume and at last an authentic translation of the *Chronic Diseases* in which we may place confidence and which will make more familiar to the rising generation of homœopathists the writings of the great founder of the school.

